# Systemic *Bartonella henselae* Infection in Immunocompetent Adult Presenting as Fever of Unknown Origin

**DOI:** 10.1155/2011/183937

**Published:** 2011-05-05

**Authors:** Thierry Zenone

**Affiliations:** Department of Internal Medicine, Centre Hospitalier Général de Valence, 179 boulevard Marechal Juin, 26953 Valence Cedex 9, France

## Abstract

Systemic clinical presentations of infection caused by *Bartonella henselae* are rare in immunocompetent adults. We report four cases with hepatic and/or splenic involvement, presenting as fever of unknown origin. We discuss diagnosis and treatment of this infection. *Bartonella henselae* serology allows an easy diagnosis of hepatosplenic involvement in cat scratch disease, a clinical picture that appears to be underrecognized.

## 1. Introduction

Cat scratch disease (CSD) caused by *Bartonella henselae* is a well-recognized benign cause of localized lymphadenopathy in immunocompetent patients, especially children [1, 2]. CSD seldom affects other organ systems, such as the liver and spleen [3]. Systemic clinical presentations are mainly described in immunodeficient adults and are less frequent in immunocompetent ones.

## 2. Cases Report

### 2.1. Case 1

A 45-year-old woman was admitted to the hospital in October 2004 because of fever (38,5°C for 3 weeks), sweats, arthralgias, and myalgias. She had lost 5 kg over a one-month period (30 kg at entry). She had no underlying diseases and was not taking medications or using alcohol or illicit drugs. There was no history of recent travel. At least 5 cats, including several kittens resided at her home. Physical examination was unremarkable; lymph nodes, liver, and spleen were not enlarged. Erythrocyte sedimentation rate (ESR) was 7 mm/h. C-reactive protein (CRP) was 13 mg/L (normal range, 0–5 mg/L). The results of WBC count and liver function tests were normal. Cultures of blood samples and urine were sterile. 

She developed right upper-quadrant abdominal pain and nausea. A CT scan of the abdomen demonstrated multiple hypodense areas within the splenic and liver parenchyma. Results of serology for *B. henselae *(indirect immunofluorescence antibody IFA) were positive for IgG (512; normal <256) and positive for IgM. Treatment with ciprofloxacin (400 mg/day IV) and gentamicin (100 mg/day IV) was started. Clinical improvement was noted. After ten days of parenteral treatment, she was treated with ciprofloxacin (1000 mg/day PO) and doxycycline (200 mg/day PO) for three weeks. At one month, a CT scan of the abdomen demonstrated mild improvement. Convalescent-phase serum samples obtained on month one were positive for IgG (1024) and IgM, and on month three were positive for IgG (1024) but negative for IgM. A CT scan of the abdomen at four months showed complete resolution of the lesions in the liver and spleen.

### 2.2. Case 2

A 27-year-old man was admitted to the hospital in May 2004 because of fever of unknown origin (38,5–40°C for four weeks) associated with fatigue, sweats, dry cough, arthralgias, and myalgias. He had lost 7 kg over a one-month period (88 kg). Before entry, he has been treated with pristinamycin 3 g/day unsuccessfully; and pulmonary radiography and thoracic CT scan yielded normal results. He had no underlying diseases and was not taking medications or using alcohol or illicit drugs. There was no history of recent travel. He had a young dog, but no cats. Physical examination was unremarkable; lymph nodes, liver, and spleen were not enlarged. ESR was 32 mm/h. CRP was 94 mg/L. The results of WBC count and liver function tests were normal. Cultures of blood samples and urine were sterile. Serologic testing were negative for Epstein-Barr virus, cytomegalovirus, hepatitis viruses A, B, and C, HIV, *Rickettsia*, *Coxiella*, *Legionella*, *Yersinia,* and *Brucella* species. A CT scan of the abdomen demonstrated multiple hypodense areas within the liver parenchyma, celiac lymphadenopathy, and homogeneous splenomegaly. Results of serology for *B. henselae* (IFA) were positive for IgG (2048; normal <256) and positive for IgM. Treatment with ciprofloxacin (400 mg/day IV) and gentamicin (160 mg/day IV) was started. Clinical improvement was noted. After ten days of parenteral treatment, he was treated with ciprofloxacin (1500 mg/day PO) and erythromycin (2000 mg/day PO) for three weeks. At one month, a CT scan of the abdomen demonstrated mild improvement. Convalescent-phase serum samples obtained on month one were positive for IgG (2048) and IgM, and on month four were positive for IgG (1024) but negative for IgM. A CT scan of the abdomen at four months was normal.

### 2.3. Case 3

A 33-year-old man was admitted to the hospital in October 2005 because of intermittent fever (39,5°C for three weeks) associated with abdominal pain. He had no underlying diseases and was not taking medications or using alcohol or illicit drugs. There was no history of recent travel. He had two cats, but he could not recall recent bites or scratches from these animals. Physical examination was unremarkable; lymph nodes, liver, and spleen were not enlarged. ESR was 41 mm/h. CRP was 140 mg/L. The results of WBC count and liver function tests were normal. Cultures of blood samples and urine were sterile. Serologic testing were negative for Epstein-Barr virus, cytomegalovirus, hepatitis viruses A, B, and C, HIV, *Rickettsia*, *Coxiella*, *Legionella,* and *Brucella* species. Echocardiography yielded normal results. A CT scan of the abdomen demonstrated three hypodense areas within the spleen parenchyma without splenomegaly and multiple celiac lymphadenopathy. A serum sample tested using an IFA assay to detect antibodies reactive with *B. henselae* was positive for IgG (2048; normal <256) and positive for IgM. Fever resolved spontaneously. However, treatment with doxycycline (200 mg/day PO) was started, and he was treated for three weeks. At one month, a CT scan of the abdomen demonstrated mild improvement. Convalescent-phase serum samples obtained on month three were positive for IgG (1024) but negative for IgM. A CT scan of the abdomen at three months showed regression of lymphadenopathy and a normal spleen.

### 2.4. Case 4

**Figure 1:**
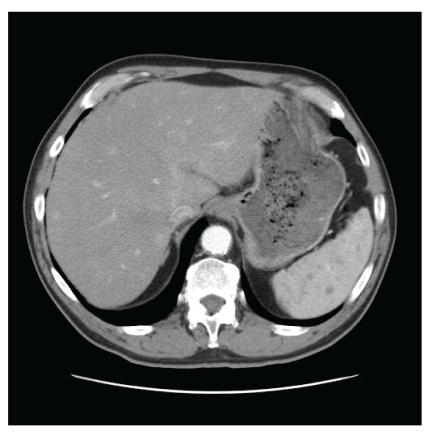
Multiple hypodense areas within the spleen parenchyma.

A 71-year-old man was admitted to the hospital in January 2009 because of fever of unknown origin (39°C for four weeks) associated with fatigue and night sweats. He had lost 4 kg over a one-month period (66 kg). He was treated for arterial hypertension and diabetes. He had two cats and described recent scratches from these animals. Physical examination was unremarkable; lymph nodes, liver, and spleen were not enlarged. ESR was 88 mm/h. CRP was 55 mg/L. The results of WBC count and liver function tests were normal. Cultures of blood samples and urine were sterile. Serologic testing were negative for Epstein-Barr virus, cytomegalovirus, hepatitis viruses B and C, HIV, *Rickettsia*, *Coxiella*, *Legionella,* and *Brucella* species. Echocardiography yielded normal results. A CT scan of the abdomen demonstrated disseminated hypodense areas within the spleen parenchyma with mild splenomegaly (Figure 1) and hypodense areas within the hepatic parenchyma. A serum sample tested using an IFA assay to detect antibodies reactive with *B. henselae* was positive for IgG (4096; normal <256) and positive for IgM. Fever resolved spontaneously, but treatment with doxycyline (200 mg/day PO) and rifampin (600 mg/day PO) was started. He was treated for three weeks with favourable outcome.

## 3. Discussion

*B. henselae* is a small gram-negative bacteria that can produce two entirely different pathologic reactions, depending on the immune status of the host: CSD, which is characterized by granulomatous nonangiogenic inflammation, and angiogenesis, in which a neutrophilic inflammatory response to bacilli located within the skin, bone, and other organs (bacillary angiomatosis) or the liver and spleen (peliosis) is seen [1]. Typically, CSD begins with a localized papule that appears 3–5 days after a cat scratch and progresses into a pustule. Tender regional lymphadenopathy develops within 1-2 weeks after inoculation. Most patients with typical CSD remain afebrile and are not systematically ill throughout the course of the disease [4]. CSD occurs primarily in children and young adults [1]. Although, recent data suggest that CSD may be more common among adults than previously thought. 

Some immunocompetent patients may have atypical CSD (systemic *B. henselae* infections), with prolonged fever (longer than three weeks), myalgia, arthralgia, and skin eruptions [5, 6]. Hepatic lesions with or without splenic lesions on ultrasonography and CT have been reported in children, and rarely in adults. Hepatosplenic involvement can be found even in patients, particularly children, with typical CSD without apparent systemic manifestations [7, 8].

Isolated splenic CSD is extremely rare, and only a few cases have been reported to date [3, 9–11]. A case of spontaneous splenic rupture as a consequence of disseminated *B. henselae* infection has been reported [12]. In many reports, the diagnosis was obtained after splenectomy had been performed [3, 10, 11]. In the case described by Ghez and coll. [9], the diagnosis of CSD was established by serological testing for *B. henselae* and splenectomy was not performed, as in our cases. Splenic CSD can easily be mistaken for splenic lymphoma, thereby leading to unnecessary splenectomy [13]. *Bartonella* serology must be achieved in case of hepatosplenic nodules with fever [2, 14]. 

A careful clinical history researching an intimate contact with a kitten associated with a specific serology and an abdominal ultrasound or CT scan for hepatosplenic involvement may follow a rapid and accurate diagnosis [15]. Radiologic features of ultrasound, computed tomography, and magnetic resonance imaging are not specific [16, 17].

Treatment of disseminated CSD in immunocompetent adult is still empirical and recovery can occur without antibiotherapy [2]. In the recent pediatric survey of Scolfaro and coll. [14], macrolides or a combination of two active antibiotics for two or three weeks leads to a rapid clinical response. Macrolides (erythromycin, azithromycin, and clarithromycin), fluoroquinolones (ciprofloxacin), trimethoprim-sulfamethoxazole, and doxycycline are frequently recommended [11]. Rifampin and gentamicin were used in the treatment of hepatosplenic CSD in children in the survey reported by Arisoy and coll [18]. In our 2 first cases, patients had febrile illness; so we used parenteral gentamicin before switching to oral therapy. In the third case, the patient did not appear acutely ill; fever spontaneously resolved; so he was treated only with oral monotherapy. The condition of many patients improves without antibiotics. Some cases may show improvement with antibiotic therapy, but it is not clear how much or how much more quickly the improvement is with therapy [6]. In the recommendations published in 2004 [4], the combination of doxycycline (100 mg PO or IV twice daily) with rifampin (300 mg PO twice daily) is proposed for treatment of complicated CSD. It was our choice in case 4. The optimum duration of antibiotic therapy for immunocompetent patients with complicated CSD has not been determined [4].

## 4. Conclusion

From the observation of four cases of systemic *B. henselae* infection between 2004 and 2009, epidemiological consequences such as underestimation in fever of unknown origin cannot be derived. However, the frequency of liver and/or splenic involvement in CSD is probably underestimated. An accurate clinical history and a reasonably wide use of the serological test may allow a rapid and accurate diagnosis, reassuring the patient and avoiding invasive and expensive diagnostic procedures, such as liver biopsy or laparotomy with splenectomy. The aim of this paper is to inform the reader that one should include systemic *B. henselae* into the differential diagnosis in cases of fever of unknown origin.
